# Effect of Membrane Fixation and the Graft Combinations on Horizontal Bone Regeneration: Radiographic and Histologic Outcomes in a Canine Model

**DOI:** 10.34133/bmr.0055

**Published:** 2024-07-29

**Authors:** Jeong-Won Paik, Yoon-Hee Kwon, Jin-Young Park, Ronald E. Jung, Ui-Won Jung, Daniel S. Thoma

**Affiliations:** ^1^ Department of Periodontology, Research Institute for Periodontal Regeneration, Yonsei University College of Dentistry, Seoul, Korea.; ^2^ Clinic of Reconstructive Dentistry, Center of Dental Medicine, University of Zürich, Zürich, Switzerland.

## Abstract

The aim of this study was to determine the effect of membrane fixation and combinations of bone substitute materials and barrier membranes on horizontal bone regeneration in peri-implant defects. Eight mongrel dogs underwent chronic buccal peri-implant dehiscence defects creation and were randomized into 4 groups: (a) deproteinized bovine bone mineral 1 (DBBM1) with a native collagen membrane (CM) (BB group, positive control group), (b) DBBM1 with native CM and 2 fixation pins (BBP group), (c) DBBM2 with a cross-linked CM (XC group), and (d) DBBM2 with cross-linked CM and 2 fixation pins (XCP group). Following 16 weeks of healing, tissues were radiographically and histomorphometrically analyzed. The total augmented area was significantly larger in the BBP, XC, and XCP groups compared to the BB group (4.27 ± 3.21, 7.17 ± 7.23, and 6.91 ± 5.45 mm^2^ versus 1.35 ± 1.28 mm^2^, respectively; *P* = 0.022). No significant difference for the augmented tissue thickness was observed among the 4 groups. The augmented tissue thickness measured at 3 mm below the implant shoulder was higher in BBP, XC, and XCP than that in BB (2.43 ± 1.53, 2.62 ± 1.80, and 3.18 ± 1.96 mm versus 0.80 ± 0.90 mm, respectively), trending toward significance (*P* = 0.052). DBBM2 and a cross-linked CM were significantly more favorable for horizontal bone regeneration compared to DBBM1 and a native CM. However, when DBBM1 and a native CM were secured with fixation pins, outcomes were similar. The addition of pins did not lead to more favorable outcomes when a cross-linked CM was used.

## Introduction

Guided bone regeneration (GBR) is a commonly encountered technique for the horizontally atrophied alveolar ridge, and this procedure is a reliable treatment modality to increase the alveolar ridge width [1]. The preferred materials for the recent GBR technique have been a combination of native collagen membrane (CM) and deproteinized bovine bone mineral (DBBM) particles. This combination has consistently yielded horizontal bone gain [2,3]. DBBM has been extensively reported as a material with slow resorption rate and high osteoconductive properties, making it one of the most frequently used and comprehensively documented bone substitutes in dental practice [4,5]. The main advantages of a native CM over DBBM are its consistent favorable results, ease of surgical handling, and relatively low risk of complications [6].

However, while the GBR technique has advanced significantly, achieving the desired results is not always guaranteed. A review study reported that the mean defect fill was 81.7% after GBR of fenestration- and dehiscence-type defects, with complete defect fill observed in only 68.5% of the cases investigated [7]. Therefore, various efforts have been made to find more predictive GBR techniques, including graft materials and barrier membranes.

There are various reasons for insufficient defect fill in the GBR procedures, and one influencing factor is the physical characteristics of native CMs. The mechanical properties of native CMs have been consistent concerns [8–10]: their inability to maintain space under flap pressure during wound closure and dimensional instability at the grafted site during the initial stage of healing. In addition, the absorption of the membrane before sufficient bone maturation or the deformation of the membrane due to pressure from overlying soft tissues [11]. The cross-linking process improves the mechanical properties of the CM and delays the degradation [12], making it a viable solution to address the limitations of native CMs. However, despite these advantages, there remain concerns regarding biocompatibility and manipulation [13,14].

Another reason for incomplete bone fill is the displacement of particulated graft materials from their initial position [15]. One method to prevent such displacement is by securing the membrane in place using pins [16,17]. However, membrane fixation using pins is technically demanding and a time-consuming procedure. There is continuing debate about the efficiency of this method in securing the membrane. Furthermore, more evidence is needed to ascertain the superiority of GBR outcomes when pins are utilized [18].

Some researchers have suggested that the CM type (i.e., native or cross-linked) may influence the dimensional stability of the grafted bone substitute materials. Friedmann et al. [19] suggested that a cross-linked CM provides adequate support for new bone formation when used with bone substitute materials without additional stabilization.

However, controlled studies investigating the effect of pin fixation depending on the type of graft combinations are lacking. This study compared 2 different combinations of barrier membranes and bone graft materials. The combinations of DBBM and CM were categorized on the basis of the type of CM (native versus chemically cross-linked), with all DBBM materials originating from bovine bone and from the same manufacturer as each barrier membrane (DBBM1 versus DBBM2).

Therefore, the aim of this study was to determine the effects of membrane fixation using 2 graft combinations on horizontal bone regeneration in peri-implant defects. The null hypothesis examined was that the regenerative outcome would not depend on the graft combinations or the use of fixation pins.

## Materials and Methods

### Ethical statements

This study was performed following the principles of “3Rs (replacement, reduction, and refinement)”, aiming to minimize animal experiment and consider animal welfare. Furthermore, this study was performed adhere to the modified guidelines of ARRIVE (Animal Research: Reporting of In Vivo Experiments) for preclinical research [20]. The Institutional Animal Care and Use Committee of Yonsei Medical Center in Seoul, Korea approved the study design including the animal selection, care, and surgical procedures (approval no: 2021-0236).

### Experimental animals

Eight mongrel dogs (aged 16 to 20 months, weighing 18 kg) were included in this study. Animals exhibited stable systemic and periodontal health throughout the study and were housed in a standard environment (room temperature at 15 to 20 °C and humidity > 30%), with unlimited access to water and a soft diet. Before the experiment, all animals underwent a 2-week adaptation period.

### Experimental materials

Thirty-two dental implants (4.0 mm in diameter and 10 mm in length; Dentium SuperLine II Implants, Suwon, Korea) were used. DBBM1 (Bio-Oss, Geistlich, Switzerland) and a native CM (Bio-Gide, Geistlich, Switzerland) were applied in groups BB and BBP. DBBM2 (Osteon Xeno, Genoss, Suwon, Korea) and a cross-linked CM (Collagen Membrane 2, Genoss, Suwon, Korea) were applied in groups XC and XCP. To minimize variability in results owing to the bone filler materials, 2 different bone substitute materials, with identical particle sizes and of the same origin (bovine bone), were selected. These materials were paired with 2 types of CMs from the same manufacturers (native versus cross-linked).

### Study design

In a split-mouth experimental design, different combinations of bone substitute materials and CMs with or without fixation pins were tested as follow:

1. DBBM 1 and a native CM (BB group, positive control group)
2. DBBM 1 and a native CM with 2 fixation pins (BBP group)
3. DBBM 2 and a cross-linked CM (XC group)
4. DBBM 2 and a cross-linked CM with 2 fixation pins (XCP group)

Allocation was performed using a randomization list (SPSS Statistics software, version20; IBM Corp., Armonk, NY, USA).

### Surgical procedure

#### Surgery 1

**Fig. 1.**
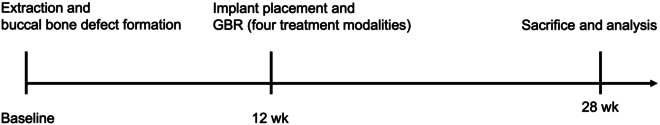
Flowchart of the study.

The surgical procedures were carried out following the administration of general anesthesia, with subsequent local anesthesia applied to the surgical site in accordance with a previously documented protocol (Fig. 1) [21]. Briefly, a gingival flap was made from premolar to the first molar area. The distal root of third premolar (P3), along with P2 and the mesial root of P4, was removed. Buccal defects were intentionally made with a round bur to develop a chronic narrow alveolar ridge in the extraction areas (10 mm × 10 mm × 5 mm). Then, the wound was closed primarily. Following the surgical procedures, the animals were administered a soft diet, and analgesics were administered for the initial 14-d postoperative period.

#### Surgery 2

**Fig. 2.**
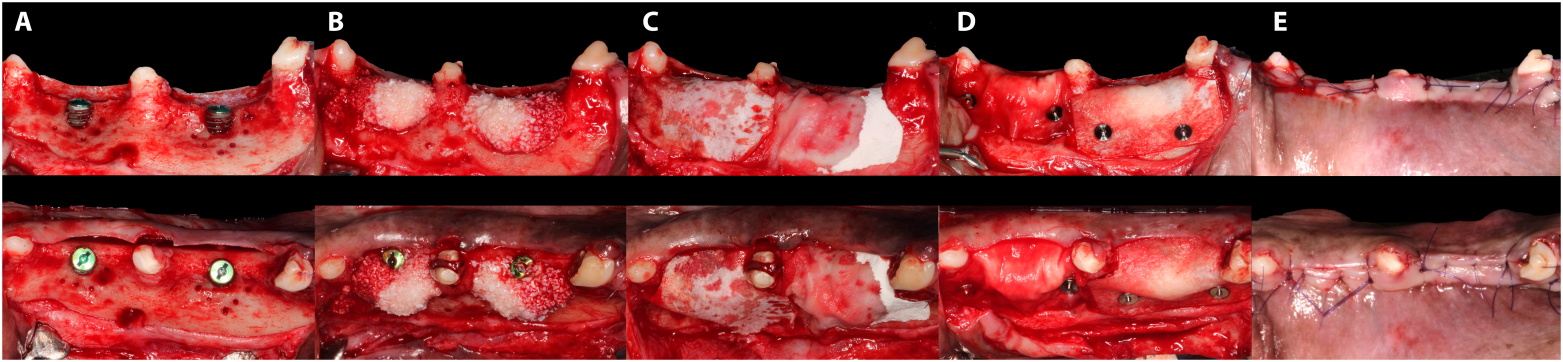
Clinical photographs of the surgical procedures. (A) Two implants are placed on each defect, leaving a buccal dehiscence (4 mm × 4 mm). (B) The defect is augmented with the particulate bone substitutes and (C) covered by 2 different membranes according to the group allocation (left: cross-linked CM; right: native CM) or (D) covered by 2 different membranes according to the group allocation (left: native CM; right: cross-linked CM) and fixed with 2 pins. (E) Primary closure is performed. Four treatment modalities are randomly selected for the peri-implant defect areas, as follows: DBBM1 and native CM (BB group), DBBM1 and native CM with pins (BBP group), DBBM2 and cross-linked CM (XC group), or DBBM2 and cross-linked CM with pins (XCP group).

After 12 weeks, the gingival flap was made to expose the healed alveolar ridge. Two implants were placed at each previous extracted site, resulting in a buccal dehiscence defect (4 mm × 4 mm). The 4 augmented modalities were assigned to the 4 peri-implant defect areas randomly. Each group received the same size CM and equal volume of bone graft material. The bone graft materials were soaked in saline and then filled into a syringe measuring 5 mm in diameter and 15 mm in length. The entire bone graft material in the syringe was placed in the defect area (total graft volume, 294 mm^3^). In the BB and XC groups, the bone defect was augmented with a bone graft material (either Bio-Oss or Osteon Xeno), and then the barrier CM (either Bio-Gide or Collagen Membrane 2, each sized 13 mm × 25 mm) was applied over the bone graft material. The bone graft material with membrane was augmented to the BBP and XCP groups using identical methods to those used in the BB and XC groups. The barrier membranes were then immobilized using 2 fixation pins at the buccal side and were pressed by the lingual flap. To facilitate primary closure, incisions were created and released as shown in Fig. 2. The animals were given soft diet and administered pain relief medication during the first 2 weeks after the surgical procedure.

### Sacrifice

After a 16-week healing period with the implants submerged, all animals were euthanized. The mandibular implant sites were dissected, preserved in 10% formalin solution, assigned unique codes, and stored for subsequent processing.

### Radiographic analysis

**Fig. 3.**
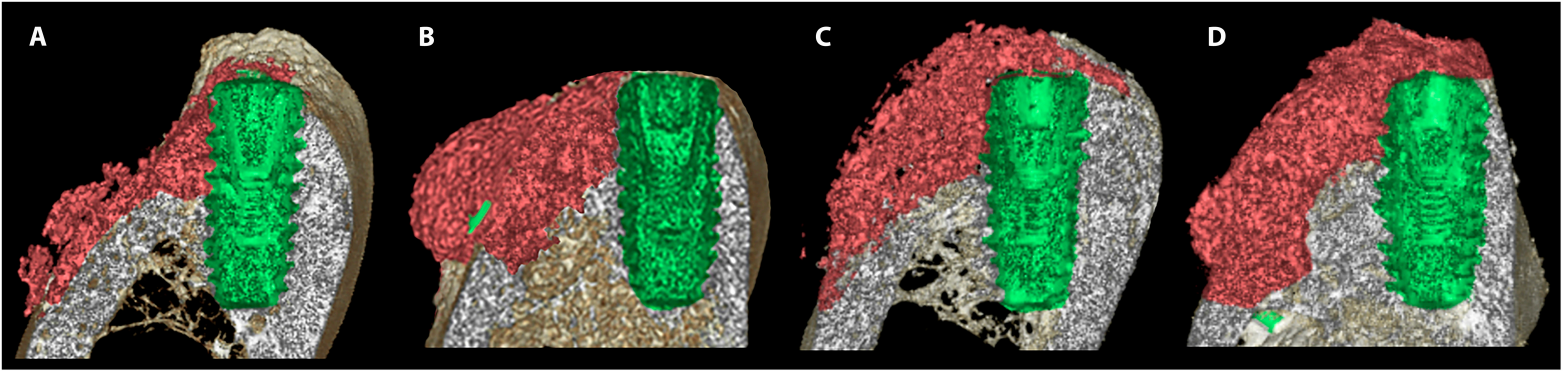
Three-dimensional reconstruction microradiographs after 16 weeks. (A) BB group: radiopaque bone substitutes (red area) scattered down along the ridge. (B) BBP group: radiopaque bone substitutes clustered above the pin (green). (C) XC group and (D) XCP group: The implants are mostly covered by radiopaque tissue, so they are not noticeable and maintain the convex shape in the coronal region.

After calibration, micro-computed tomography (micro-CT; SkyScan 1173, SkyScan, Belgium) was conducted on the specimens using with a resolution of 16 μm (130 kV and 60 μA). The scanned data were used to generate 3-dimensional images utilizing the NRecon software (Bruker Micro-CT) (Fig. 3).

### Histomorphometric analysis

Each specimen was embedded in paraffin, and consecutive coronal sections with 30 μm in thickness sequentially sliced along the center. The central section of each block was selected and stained with Masson’s trichrome solution and hematoxylin and eosin staining. The analyses were conducted by a sole proficient investigator (Y.-H.K.) using image analysis software (Case Viewer, 3D HISTECH; and Photoshop CC, Adobe, San Jose, CA, USA).

The surface areas of the total augmented area (TAA; in square millimeters) and the area attributed to each composition within a region of interest (3 mm wide × 3 mm high) from the implant shoulder were recorded as follows:

• TAA
• New bone area (NBA)
• Residual bone graft material area (RBA)
• Fibrovascular tissue area (FVA)

The following vertical distances were also measured (in millimeters):

• I-Bc, the distance from the implant shoulder to the most coronal point of the bone crest
• I-fBIC, the distance from the implant shoulder to the first bone contact (first bone-to-implant contact)

Horizontal linear measurements perpendicular to the longitudinal axis of the implant were conducted on the buccal aspect, yielding the following results (in millimeters):

• Augmented tissue thickness (AGT), the measurement from the implant surface to the barrier membrane at depths of 1, 2, 3, and 4 mm apical to the implant shoulder (identified as AGT-1, AGT-2, AGT-3, and AGT-4, respectively).

To determine the volume stability during the simultaneous use of a different graft combinations of DBBM and CM, with or without fixation pins, TAA was set as the primary outcome. The remaining measures were considered secondary outcomes.

### Statistical analyses

The sample size was calculated using G*power 3 software (Franz Faul, Christian-Albrechts-Universität Kiel, Kiel, Germany). Assuming an effect size of 0.65, an α error of 0.05, and a power of 80%, the total sample size required was determined to be 32 (4 samples per animal), accounting for the 4 groups and attrition ratio.

Data are reported as means ± SD. Group comparisons were performed using the Kruskal–Wallis test, followed by post hoc pairwise comparisons with Bonferroni’s correction for multiple testing. Statistical analysis was performed with SPSS Statistics software (Chicago, IL, USA), with a significance threshold set at *P* < 0.05.

## Results

### Clinical observations

One animal exhibited postoperative swelling and bleeding reaction at all implant sites, while another experienced soft tissue exposure at the BBP site. Surgical wound healing at all implant sites in the remaining animals was uneventful throughout the experimental period, without any complications such as severe swelling, wound exposure, or bleeding.

### Radiographic findings

The 3-dimensional micro-CT images are presented in Fig. 3. In the BB group, radiopaque tissue partially covered the peri-implant defects; however, albeit at all sites, 1 to 3 implant threads remained unmineralized. Bone substitute particles were scattered around the defect sites at the CM site. The peri-implant defects in the BBP group were also filled with radiopaque tissue. Fixture thread exposure was observed in only 2 of the 10 animals. Compared with the BB group, in the BBP group, the grafted bone substitutes were mostly distributed above the pin, with few scattered particles visible below the pin. Three implants in the XC group were not noticeable because they were completely covered by radiopaque tissue, and the other defects were also covered so that the fixture threads were not exposed, except for the dog that had inflammation. The XCP group showed findings very similar to those of the XC group and maintained a convex shape in the coronal region (Fig. 3).

### Histologic findings and histomorphometric analysis

**Fig. 4.**
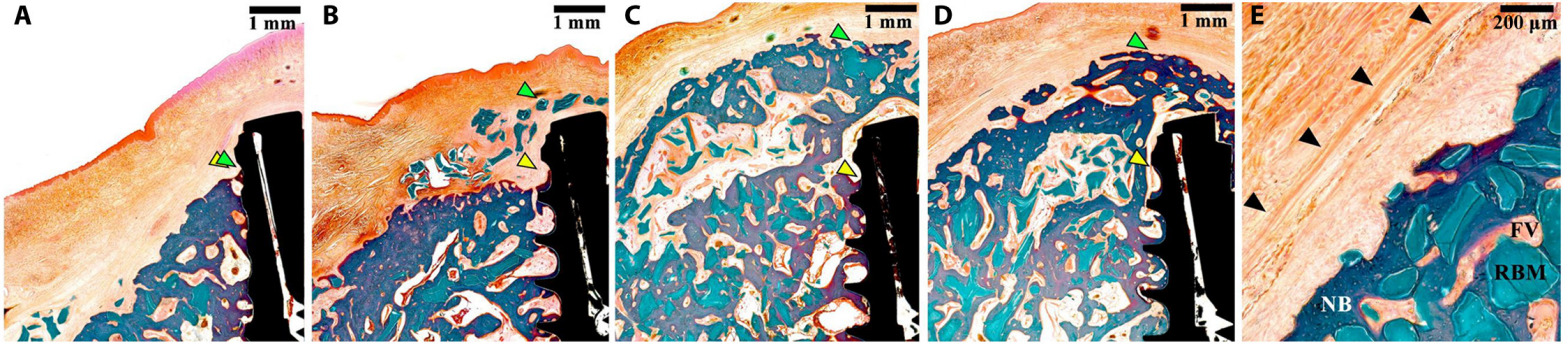
Histological views after 16 weeks. (A) BB group, (B) BBP group, (C) XC group, and (D) XCP group. (E) Arrowheads in the magnified view indicate the residual CM following 16 weeks of healing in the XCP group (Masson’s trichrome stain). RBM, residual bone graft material; FV, fibrovascular tissue; NB, new bone.

Most specimens demonstrated bone regeneration along the buccal aspect of the implant, resulting in a convex ridge contour. The XC and XCP groups demonstrated a layered structure of dense collagen fibers with the remaining membranes, whereas the BB and BBP groups demonstrated greater membrane resorption (Fig. 4).

**Fig. 5.**
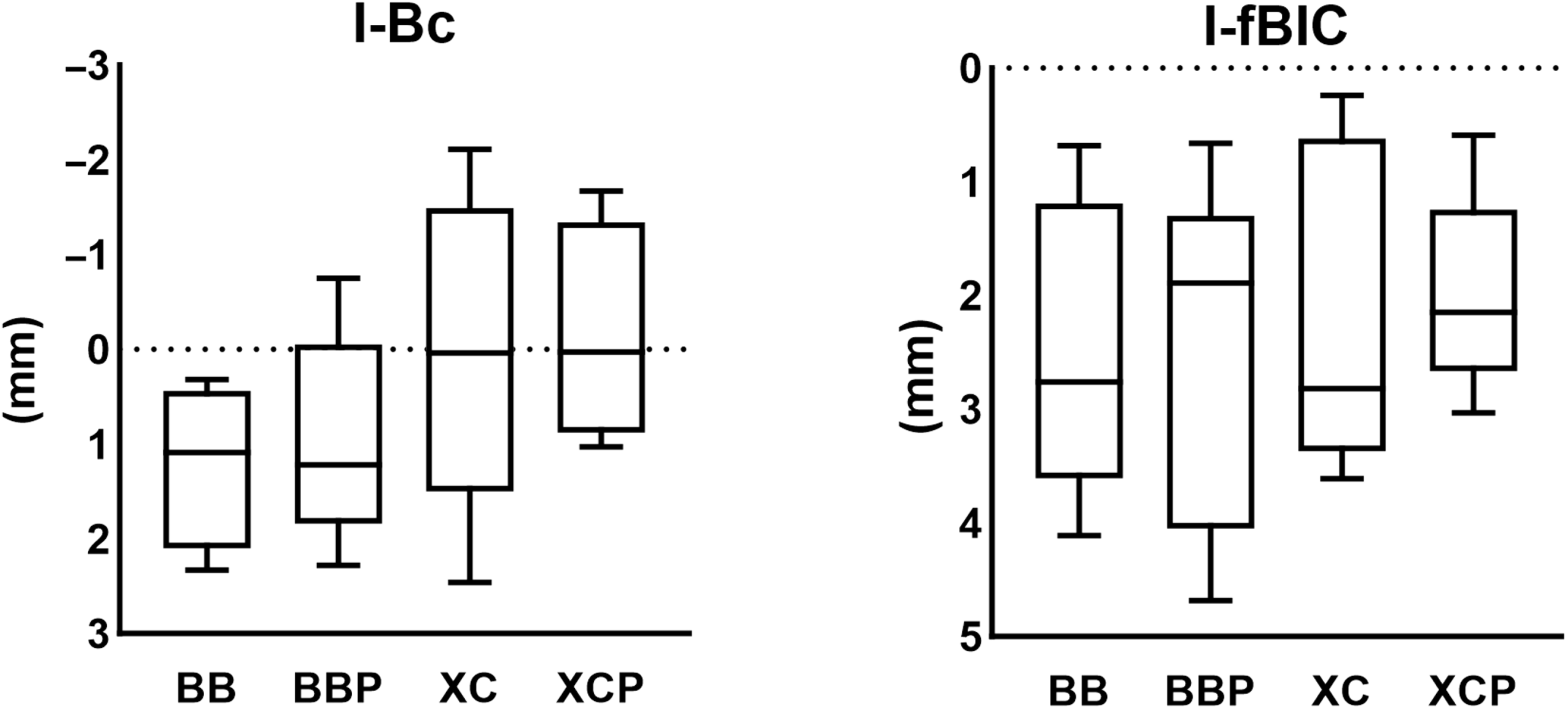
Vertical measurements. I-Bc, the distance from the implant shoulder to the most coronal point of the bone crest; I-fBIC, the distance from the implant shoulder to the first bone contact (first bone-to-implant contact). There is no significant difference among the 4 groups (*P* < 0.05).

**Fig. 6.**
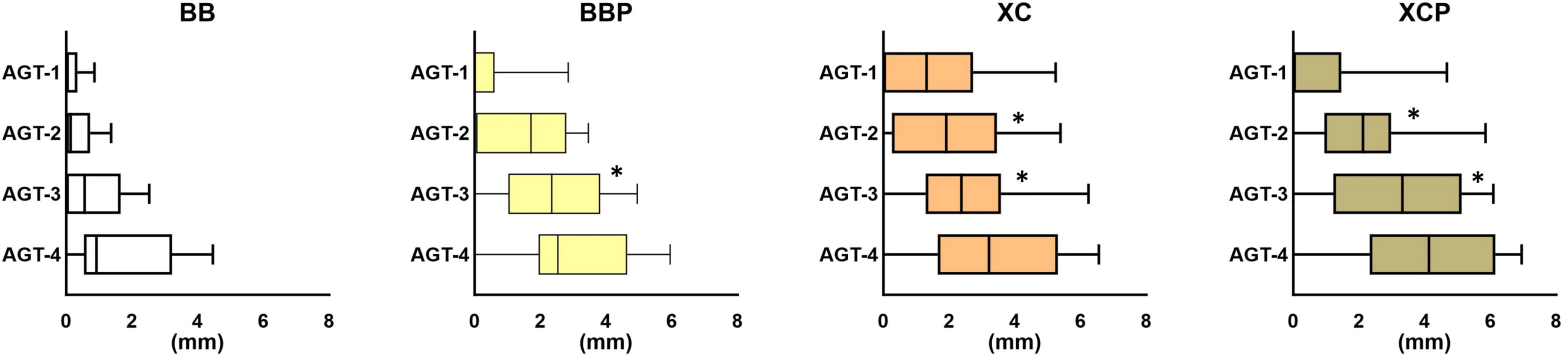
Horizontal measurements. AGT, augmented tissue thickness at 1, 2, 3, and 4 mm apical to the implant shoulder (identified as AGT-1, AGT-2, AGT-3, and AGT-4, respectively). Asterisks (*) denote significantly different compared with the BB group in the post hoc test (*P* < 0.05).

Histomorphometric measurements are displayed in Table and Figs. 5 and 6.

**Table.** Histomorphometric analysis measurements (mean ± standard deviation, mm^2^)

	TAA	NBA	RBA	FVA
BB	1.35 ± 1.28	0.35 ± 0.68	0.16 ± 0.26	0.84 ± 0.80
BBP	4.27 ± 3.21^a^	1.12 ± 1.50	0.65 ± 0.64	2.50 ± 1.64^a^
XC	7.17 ± 7.23^a^	1.50 ± 2.73	1.47 ± 1.70	4.20 ± 3.19^a^
XCP	6.91 ± 5.45^a^	1.70 ± 2.86	1.02 ± 1.12^a^	4.19 ± 2.17^a^

TAA, NBA, RBA, and FVA were measured within a region of interest (3 mm in width and 3 mm in height) from the implant shoulder. BB group, DBBM1 and native CM; BBP group, DBBM 1 and native CM with pins; XC group, DBBM 2 and cross-linked CM; XCP, DBBM 2 and cross-linked CM with pins.

^a^ Significantly different from BB group (*P* < 0.05).

#### Area measurements

The TAA showed a significant increase in the BBP, XC, and XCP groups than in the BB group (4.27 ± 3.21, 7.17 ± 7.23, and 6.91 ± 5.45 mm^2^ versus 1.35 ± 1.28 mm^2^, respectively; *P* = 0.022). This was predominantly based on a significantly larger FVA in the BBP, XC, and XCP groups than that in the BB group (2.50 ± 1.64, 4.20 ± 3.19, and 4.19 ± 2.17 mm^2^ versus 0.84 ± 0.80 mm^2^; *P* = 0.023). However, there were no statistically significant differences observed in the NBA and RBA among the 4 groups (*P* > 0.05).

#### Linear measurements

The defect heights (I-fBIC and the I-Bc) were lower in the BBP, XC, and XCP groups than those in the BB group; however, the difference was not statistically significant (*P* > 0.05).

Horizontally, AGT did not differ among the 4 groups, although the results at 3 mm below the implant shoulder are on the border of the significance level (*P* = 0.052). The post hoc test results revealed that the BBP, XC, and XCP groups were significantly greater than the BB group at the 3-mm level (2.43 ± 1.53, 2.62 ± 1.80, and 3.18 ± 1.96 mm versus 0.80 ± 0.90 mm, respectively). Moreover, the XC and XCP groups showed a significant difference even at the 2-mm level (2.07 ± 1.73 and 2.22 ± 1.68 mm versus 0.36 ± 0.47 mm, respectively).

## Discussion

This study assessed the effect of membrane fixation on horizontal bone regeneration in peri-implant dehiscence defects using different combinations of DBBM and CMs in a canine model. The results showed that DBBM2 and a cross-linked CM (with or without fixation pins) group showed significantly more favorable histological outcomes for horizontal bone regeneration compared to DBBM1 and a native CM. However, when fixation pins were used with DBBM1 and a native CM, similar outcomes were observed. The addition of pins did not result in more favorable outcomes when a cross-linked CM was used. Thus, the null hypothesis was partially disproved.

The principle of GBR relies on maintaining a contained space around a bone defect to promote blood clot stabilization using an occlusive barrier membrane [22]. In other words, the immobilization and stability of the barrier membrane and the grafted bone material are the key factor for space maintenance. Mir-Mari et al. [11] reported that although they closed the flaps without tension in all surgical cases in their study, tissue pressure at the crestal area of the grafted site inevitably occurred during suturing; they also reported that the fixation using pins enhanced the adaptation of the barrier membrane and immobilization of the grafted material in the desired coronal position. However, that study primarily measured the volume of grafted material before and after suturing; therefore, it did not address the effects of pin fixation during the healing process. The clinical implications and benefits of using fixation pins in clinically relevant models have not been investigated.

An animal study examining the effect of pin using a native CM has been reported [17]. In the conclusion, the authors reported no significant differences in GBR outcomes depending on pin fixation; however, the use of a box-shaped contained defect model in the experiment may introduce potential discrepancies from clinical practice. Most studies to date have experimented box-shaped osseous defects created using burs in healed alveolar ridges, as mentioned. However, these acute osseous defects have distinct bony walls not only at the mesial and distal walls but also at the base, which may render them more favorable in terms of avoiding the scattering of bone grafting materials. The osseous defect configuration used in this study attempted to mimic a clinical situation frequently encountered in implant dentistry, featuring a narrow crest and a broad apical alveolar ridge. This results in a buccal peri-implant dehiscence defect after fixture insertion. These experimental model and study were designed to provide insights into the efficacy of GBR in clinically relevant anatomical situations.

The most notable finding of this study was that TAA was significantly larger in the BBP, XC, and XCP groups than that in the BB group *(P* = 0.022). Among the 4 groups, AGT exhibited no statistically significant differences; however, it did indicate a strong trend (*P* = 0.052) that did not reach statistical significance. The BBP, XC, and XCP groups had greater AGT values compared to the BB (2.43 ± 1.53, 2.62 ± 1.80, and 3.18 ± 1.96 mm versus 0.80 ± 0.90 mm, respectively). The post hoc test results showed that the BBP, XC, and XCP groups exhibited significantly higher values compared to the BB group at the 3-mm level (*P* = 0.038, *P* = 0.038, and *P* = 0.015, respectively). The XC and XCP groups showed a significant difference even at the 2-mm level (*P* = 0.05, and *P* = 0.01, respectively).

These results suggest that using 2 pins to secure the membrane in the native CMs prevents the displacement of the particulate bone substitute materials, whereas cross-linked CMs show similar favorable results both with and without pins. These results could be explained by slower absorption rate and the mechanical properties of the cross-linked CMs. Although the DBBMs used have the same origin (bovine bone) and particle size, they were from a different manufacturer. The 4 experimental groups were formed by combining 2 types of CM (native versus cross-linked) with matching bone substitutes from the same manufacturer. In addition, the primary outcome was the volume stability effect to maintain the bone substitutes without scattering, rather than the new bone formation. Therefore, we cautiously suggest that the effect of the barrier membrane may play a more pronounced role than the DBBM material used.

It should be noted that the most critical area for horizontal GBR at peri-implant defects is not located at the apical region but at the implant shoulder level. The lack of statistical significance at levels 1 and 2 (AGT), which are considered more clinically relevant, and the significance observed only in the post hoc test at level 3 raise doubts about the overall benefit of using fixation pins. Despite being observed in the post hoc test results, the XC and XCP groups showed a significant difference even at the 2-mm level.

Even native CMs demonstrate excellent biocompatibility and easy clinical handling. Inherent drawbacks associated with this type of membrane include an unpredictable degradation period and a lack of rigidity for space maintenance [5]. Cross-linked CMs, which were developed to overcome the shortcomings of native CMs, offer superior physical properties and an extended resorption period [23,24]. These advantages are, to some extent, limited by the drawback of having a 30% higher membrane exposure rate than that of native CMs, as documented in a systematic review [25]. Recently, various cross-linking process have been developed to enhance biocompatibility and facilitate manipulation. Consequently, many previously reported problems have been substantially reduced. These improved membranes are commonly utilized in clinical practice [14,19].

The histological findings of this study presented that DBBM2 and cross-linked CM groups exhibited a dense collagen fiber layer after 16 weeks of healing, indicating prolonged structural integrity. Extended biodegradation of cross-linked CMs may enhance the regenerative potential of GBR interventions [9,14,26]. Histomorphometric analyses showed that no significant differences between the XC and XCP groups in area and linear comparisons (vertical and horizontal). In other words, when using cross-linked CM, a similar level of augmented volume was achieved in the coronal region, irrespective of the use of pins. It is assumed that the rigidity of cross-linked CMs provides resistance against the pressure exerted by the surrounding soft tissues, thereby mitigating the risk of displacement or collapse of the graft materials. Friedmann et al. [19] compared the horizontal bone augmentation between DBBM + native CM + pins and DBBM + cross-linked CM. They reported a significantly larger tissue area in the group that used cross-linked CM without pins than native CM with pins. They emphasized that the membrane properties play a more crucial role than fixation. Cross-linked CMs without pins maintain a greater volume of augmented tissue than native CMs with fixation [19]. Hence, it is presumed that the mechanical properties of the barrier membrane play a critical role in the volumetric stability of horizontal bone regeneration.

However, this study did have some limitations; namely, we included a relatively small number of dogs and a relatively homogeneous sample including 8 mixed-breed dogs. This may limit the strength and generalizability of our findings. An increase in the number of sites/animals would further strengthen our findings.

Another limitation of this study is its focus on comparing scaffolds for bone regeneration, with somewhat limited consideration of other critical factors for the success of GBR such as growth factors. Furthermore, recent research has been exploring tissue engineering approaches that can enhance bone regeneration with minimally manipulation of isolated cells during surgery [27,28]. Considering the importance of scaffolds in the clinical application of these tissue engineering approaches, this study aimed to establish a protocol for optimizing the surgical application of osteoconductive scaffolds. It is anticipated that advanced research incorporating tissue engineering will continue following the establishment of the surgical protocol.

In future research, it is necessary to conduct comparative studies with a larger sample size and evaluate long-term outcomes to establish the findings of this study more firmly. Furthermore, tissue engineering approach aimed at accelerating bone regeneration and enhancing bone quality should also be considered. The ultimate goal of these studies is to develop the optimal GBR protocol in a clinical practice.

Despite the limitations of this study, DBBM2 and a cross-linked CM showed significantly favorable histologic outcomes for horizontal bone regeneration compared to DBBM1 and a native CM. However, when DBBM1 and a native CM were secured with fixation pins, it demonstrated similar outcomes. The addition of pins did not lead to more favorable outcomes when a cross-linked CM was used.

### Conclusion

DBBM2 and a cross-linked CM were significantly more favorable for horizontal bone regeneration compared to DBBM1 and a native CM. Membrane fixation using pins is beneficial and effective for native CMs. However, securing cross-linked CM using pins did not result in further benefits based on histologic and radiographic outcome measures.

## Data Availability

The data that support the findings of this study are available from the corresponding author upon reasonable request.
